# AllerHunter: A SVM-Pairwise System for Assessment of Allergenicity and Allergic Cross-Reactivity in Proteins

**DOI:** 10.1371/journal.pone.0005861

**Published:** 2009-06-10

**Authors:** Hon Cheng Muh, Joo Chuan Tong, Martti T. Tammi

**Affiliations:** 1 Department of Biological Sciences, National University of Singapore, Singapore, Singapore; 2 Data Mining Department, Institute for Infocomm Research, Singapore, Singapore; 3 Department of Biochemistry, National University of Singapore, Singapore, Singapore; 4 Department of Microbiology, Tumor and Cell Biology, Karolinska Institutet, Stockholm, Sweden; University of East Piedmont, Italy

## Abstract

Allergy is a major health problem in industrialized countries. The number of transgenic food crops is growing rapidly creating the need for allergenicity assessment before they are introduced into human food chain. While existing bioinformatic methods have achieved good accuracies for highly conserved sequences, the discrimination of allergens and non-allergens from allergen-like non-allergen sequences remains difficult. We describe AllerHunter, a web-based computational system for the assessment of potential allergenicity and allergic cross-reactivity in proteins. It combines an iterative pairwise sequence similarity encoding scheme with SVM as the discriminating engine. The pairwise vectorization framework allows the system to model essential features in allergens that are involved in cross-reactivity, but not limited to distinct sets of physicochemical properties. The system was rigorously trained and tested using 1,356 known allergen and 13,449 putative non-allergen sequences. Extensive testing was performed for validation of the prediction models. The system is effective for distinguishing allergens and non-allergens from allergen-like non-allergen sequences. Testing results showed that AllerHunter, with a sensitivity of 83.4% and specificity of 96.4% (accuracy = 95.3%, area under the receiver operating characteristic curve AROC = 0.928±0.004 and Matthew's correlation coefficient MCC = 0.738), performs significantly better than a number of existing methods using an independent dataset of 1443 protein sequences. AllerHunter is available at http://tiger.dbs.nus.edu.sg/AllerHunter

## Introduction

Allergic diseases represent one of the most common chronic health problems in recent years, affecting more than 20% of the general population [1]. Food allergy affects 4% [2] of the US population, while asthma and atopic dermatitis were reported in 10% [3] and 15% [4] of the children worldwide. Allergic responses result from adverse immunologic reaction to causative agents known as allergens that are otherwise innocuous in nature. Type I hypersensitive reaction is initiated when an allergen interacts with IgE antibodies on the mast cells or basophils, resulting in the release of inflammatory mediators [5]. This may be followed by a late-phase reaction characterized by the influx of T-cells, eosinophils and monocytes. Symptoms of the disease vary greatly, and include asthma, conjunctivitis, dermatitis, rhinitis, as well as the more severe anaphylaxis.

The number of modified proteins in foods, therapeutics and bio-pharmaceuticals [6], [7] are increasing rapidly creating the need for assessing potential allergenicity before new proteins are brought into contact with humans. The current joint recommendation by the World Health Organization (WHO) and Food and Agriculture Organization (FAO) is a scheme based on a decision tree which compares local sequence similarity of a query protein against known allergenic proteins [8]. In addition to biological tests on the protein of interest, two decision criteria have been proposed for the assessment of allergenic potential: identity of six or more contiguous amino acids, or minimum 35% sequence similarity over a window of 80 amino acids. Although these criteria are useful in some cases [9], the precision is low for methods solely relying on the six amino acid rule [10]. More sophisticated bioinformatic tools for detecting motifs among allergenic sequences have been described, including the use of k-Nearest-Neighbor (kNN) classifiers [11], linear/quadratic Gaussian classifiers [12], Fourier transforms [13], allergen-representative peptides (ARPs) [14], global protein descriptors [15] and hybrid techniques [16]. While these systems are effective for high similarity allergen sequences, they are less effective when the overall similarity is low. Moreover, similarity in protein folds does not necessarily lead to cross-reactivity [17], and the discrimination of allergens and non-allergens from allergen-like non-allergen sequences remains difficult. Hence more sophisticated methods are necessary for discriminating such sequences.

In this paper, we present AllerHunter, an allergenicity prediction system that appears to be capable of detecting motif- or domain-sized similarities in novel proteins even when overall sequence similarity with known allergens is low. The system is based on the integration of FAO/WHO evaluation scheme and a statistical learning method, known as SVM-pairwise, which has been very successful for remote protein homology detection [18], [19]. The system is rigorously trained and tested using 1,356 known allergens and 13,449 putative non-allergens. The effectiveness of SVM-pairwise method in detecting the potential allergenicity and allergic cross-reactivity in protein sequences is evaluated. It outputs a likelihood score and the corresponding accuracy level. This, in corporation with the FAO/WHO evaluation scheme, allows a more comprehensive assessment of potential allergenicity in proteins.

## Materials and Methods

### Dataset

The dataset consists of 14,805 (1,356 allergens and 13,449 putative non-allergens) sequences. Known allergen protein sequences were extracted from GenBank [20], Swiss-Prot's Allergen Index [21], Allergome [22], the Food Allergy Research and Resource Program (FARRP) Protein AllergenOnline Database [23], the Structural Database of Allergenic Proteins (SDAP) [24] and the Allergen Nomenclature database of the International Union of Immunological Societies (IUIS) [25]. An initial list of protein sequences unlikely to be associated with allergy was generated by extracting all protein sequences from Swiss-Prot with the exception of entries containing text strings ‘allergen’, ‘allergy’, ‘atopy’ or derivatives thereof in the annotation [12]. The resulting putative non-allergen dataset of 217,171 protein sequences was divided into 8,449 allergen-like putative non-allergens (APN) and 208,722 divergent putative non-allergens (DPN) based on the criteria of identity ≥30% and coverage ≥50% [17], [26] with known allergens. From this list, one tenth of all 8,449 APNs and 5,000 randomly selected DPNs, together with the known allergen sequences, were randomly selected for independent testing. The remaining sequences were randomly divided into five sets for five-fold cross-validation. The training and testing was carried out five times, each using one distinct set for testing and the remaining four sets for training. Performance of the optimized model was assessed using the independent data set.

### Pairwise similarity scores as feature vectors

**Figure 1:**
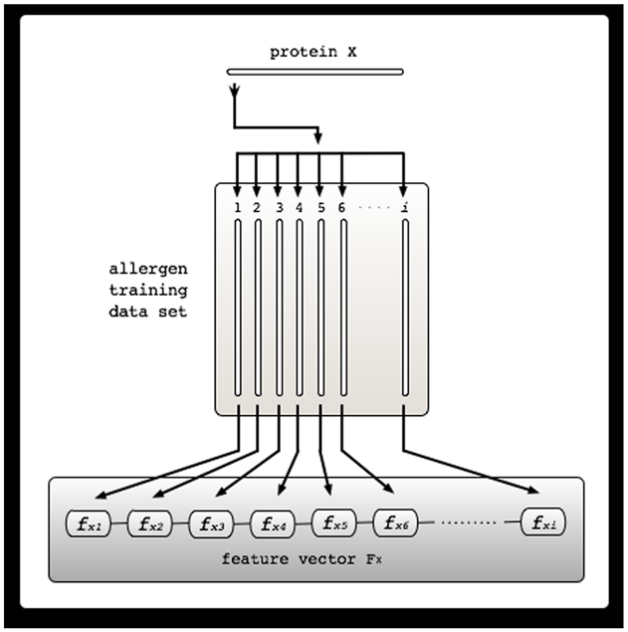
An iterative pairwise sequence similarity training scheme used for constructing a protein's feature vector. Feature vector corresponding to a particular protein X is F_X_ = f_X1_, f_X2_, …, f_Xi_, where *i* is the total number of allergens in the training data set and f_Xi_, is the Smith-Waterman alignment score of sequence X against the *i*th allergens in the training dataset.

An iterative pairwise sequence similarity training scheme was used for constructing a protein's vector representation. The method involves decomposing a given sequence into its similarity with a list of known allergen and putative non-allergen sequences. First, the entire training set sequences was vectorized using the Smith-Waterman algorithm [27]. The BLASTP algorithm as implemented in WU BLAST 2.0 [28] was used to compute the pairwise similarity scores of each protein against all other members of the training set. For each protein X, the input vector is defined as F_X_ = f_X1_, f_X2_, …, f_Xn_, where n is the total number of proteins in the training dataset and f_Xi_ is the E-value of the Smith-Waterman score between sequence X and the *i*th training set sequence (Figure 1) [18]. The BLOSUM62 substitution matrix was used, with gap opening penalty and extension penalty of 11 and 1, respectively.

### Support vector machine

Support vector machines (SVM) [29], [30] are statistical learning methods based on the structural risk minimization principle [31]. The method employs a kernel function to project input vectors into a high-dimensional feature space, and selecting a hyperplane within the space that maximizes the separation of the positive (allergens) and negative (non-allergens) examples. The base SVM kernel is normalized so that each vector has length 1 in the feature space: 
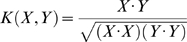
(1)This kernel 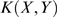 is then transformed into a radial basis kernel 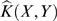 as follows: 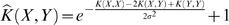
(2)where the σ is the median Euclidean distance (in feature space) from any positive training example to the nearest negative example. The constant 1 is added to the kernel in order to translate the data so that the separating hyperplane passes through the origin [18], [19]. The method can then classify an unlabeled example by mapping it into the feature space and identifying on which side of the separating plane the example is located. The SVM algorithm, which provides the framework of the SVM-pairwise method, has been implemented in AllerHunter using LIBSVM [32].

### Performance measures

Five-fold internal cross-validation was performed to assess to quality of the model (). In *k*-fold cross-validation, *k* random, (approximately) equal-sized, disjoint partitions of the sample data are constructed, and a given model is trained on (*k*−1) partitions and tested on the excluded partition. The results are averaged after *k* such experiments, and the observed error rate may be taken as an estimate of the error rate expected upon generalization to new data.

The performance of AllerHunter was assessed using sensitivity (SE), specificity, (SP), accuracy (ACC), Matthew's correlation coefficient (MCC) and the area under the Receiver Operating Characteristic Curve (AROC). SE = TP/(TP+FN) and SP = TN/(TN+FP), indicate percentages of correctly predicted allergens and non-allergens, respectively. ACC = (TP+TN)/(TP+TN+FP+FN) indicates the percentage of correctly predicted sequences. TP (true positives) stands for known allergens and TN (true negatives) for non-allergen protein sequences. FN (false negatives) denotes known allergens predicted as non-allergens, and FP (false positives) represents non-allergens predicted as allergens. The AROC and MCC, which are used as measures of the quality of the prediction, are defined as follows: 
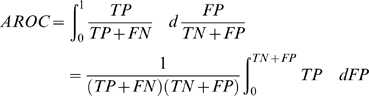
(3)

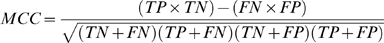
(4)The MCC returns a value between −1 and 1: MCC = 1 for 100% accuracy, MCC = 0 for 50% accuracy and MCC = −1 for 0% accuracy. The MCC was used for comparison of performances with different predictions.

## Results

The robustness of AllerHunter has been estimated for 5-fold cross-validation. The results indicate that the system is stable (AROC = 0.928±0.004), rendering it suitable for predictions on the test dataset. The performance of AllerHunter was next assessed using an independent dataset of 129 known allergens and 1314 putative non-allergens (826 APNs and 488 DPNs). The results indicate that, overall, SVM-pairwise is suitable for discriminating allergen protein sequences from the background with good accuracy (SE = 83.7%, SP = 96.4%, ACC = 95.3%, MCC = 0.738). The system is capable of discriminating allergens from both allergen-like sequences (SP = 98.3%; or 812 out of 826 APNs) and non-allergen-like sequences (SP = 93.2%; all 455 out of 488 DPNs).

### Performance on Swiss-Prot

The efficacy of the system was then evaluated using 217,551 proteins from Swiss-Prot [21], to determine if the approach is consistent with other existing studies. A total of 3,537 or 1.6% of proteins in Swiss-Prot is predicted as allergen protein sequences. This is consistent with the 2.9% (4,943) of 168,128 Swiss-Prot protein entries from SVM global descriptor approach [15], 4.0% (4,093) of 101,602 Swiss-Prot protein entries from a motif-based method [33] and 3.5% (4,768) of 135,850 Swiss-Prot protein entries from Fourier transform [13].

### Comparison with existing methods

A number of allergenicity prediction systems have been reported by various groups and made available to the public. To benchmark the performance of SVM-pairwise against these systems, the independent dataset of 1,443 sequences was used to evaluate four available techniques – i) AlgPred, which combines several prediction methods such as SVM using amino acid and dipeptide composition, motif-based prediction and IgE epitope mapping [16]; ii) APPEL, an SVM-based technique using the physicochemical properties of amino acids [15]; DASARP, based on sequence similarity scores derived from allergen-representative peptides (ARPs) [14]; and FAO/WHO evaluation scheme based on identity of six or more contiguous amino acids, or minimum 35% sequence similarity over a window of 80 amino acids [34].

**Figure 2:**
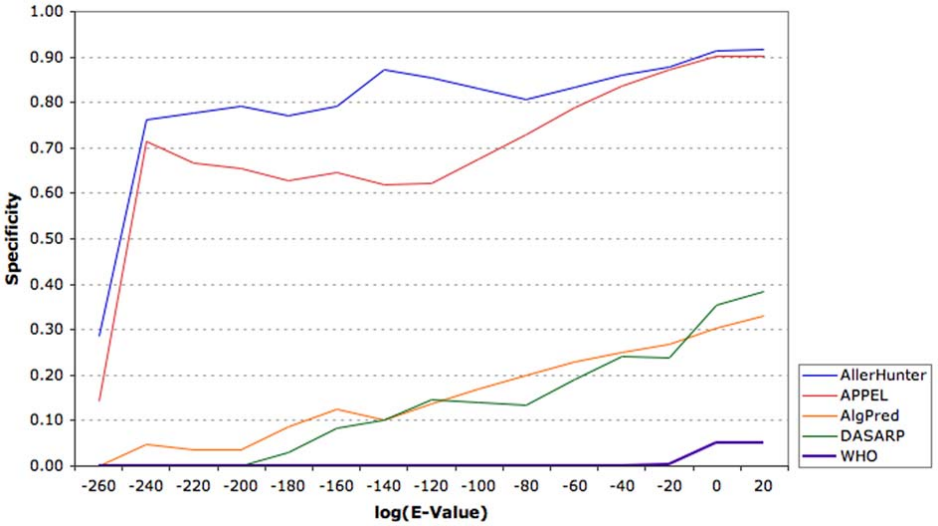
Comparison of SVM-pairwise's performance against existing systems. A cumulative plot of specificity against log(E-value) is shown; indicating that SVM-pairwise is more capable of differentiating allergen-like non-allergens that other reported systems.

As illustrated in Table 1, the SVM-pairwise method (SE = 83.7%, SP = 96.4%, ACC = 95.3%, MCC = 0.738) consistently outperforms all existing techniques tested in this study. The predictive performances of AlgPred (MCC = 0.201) and DASARP (MCC = 0.298) are low, suggesting that these systems may be over-optimized for positive prediction. Although the FAO/WHO evaluation scheme has the highest sensitivity of 97.8%, it also has a low specificity (27.9%). This is consistent with existing reports on its inherent limitations that its precision may be too low to be of practical use [35]. Moreover, the computed MCC for this scheme is 0.001, indicating near random prediction. The systems were evaluated on their abilities to discriminate allergens from allergen-like sequences and non-allergen-like sequences. As illustrated in Table 1, the SVM-pairwise approach developed in this study has a high specificity of 91.1% and 100.0% for both sets of APN and DPN protein sequences. An accumulative plot of SP against log(E-values) (Figure 2) showed that SVM-pairwise significantly outperforms all existing techniques with increasing sequence similarity, i.e., lower log(E-value). These results indicate that SVM-pairwise is suitable for predicting potential cross-reactivities in novel proteins that lack high sequence similarity to any of the known allergen sequences, and is also capable of discriminating allergens and non-allergens from allergen-like non-allergen sequences. When integrated with the FAO/WHO evaluation scheme, the system, through the provision of likelihood scores and the corresponding performance measures, allows for more comprehensive assessment of the potential allergenicity of proteins.

**Table 1:** Comparison of the performances between SVM-pairwise and state-of-the-art techniques using an independent dataset of 1,443 sequences.

Method	SE (%)	ACC (%)	SP (%)		MCC
			All	All APNs	
FAO/WHO	97.8	20.9	27.9	0.03	0.001
AlgPred	92.2	46.4	75.9	28.1	0.201
DASARP	91.0	94.3	85.9	33.2	0.298
APPEL	81.4	92.7	96.4	89.6	0.641
SVM-pairwise	83.7	95.3	96.4	98.3	0.738

The specificity (SP) of the system was assessed using i) all putative non-allergens, ii) allergen-like putative non-allergens (APN) iii) 100 APN sequences with lowest E-values and iv) divergent putative non-allergens (DPN), respectively.

## Discussion

It has been reported that similarity in protein folds does not necessary lead to cross-reactivity between two allergens [17]. Hence, a single threshold of similarity might not be effective for assessing all potential allergenicity. In this aspect, AllerHunter considers a profile of pairwise similarities to both allergen and non-allergen sequences for inferring the potential allergenicities and allergic cross-reactivities in protein sequences. The adopted feature vectors, which consist of pairwise sequence similarity scores of allergenic and non-allergenic protein sequences, represent the homology among proteins that are known to elicit allergic responses. Such a profile of alignment scores summarizes the differences between a given sequence and a given family of allergen proteins. It has the potential to identify remote relationships in distantly related protein sequences, which may not be effectively modeled by existing sequence-based computational strategies [18], [19]. Given the high diversity of allergen sequences, the proposed encoding scheme amplifies similarities and differences between allergens and non-allergens through the use of a series of sequence identities instead of a universal weighting scheme employed by existing techniques. We have shown that the SVM-pairwise method consistently outperforms current state-of-the-art algorithms. To date, the general characteristics of allergens such as structural, functional or biochemical properties that explain their ability to elicit allergic responses remain unclear [36]. Given the complex nature of allergic cross-reactivity, the methodology proposed herein may be useful for both the analysis of allergenicity and the better understanding of the biological basis of allergy.
